# Long-term survival with surgery for metachronous retroperitoneal lymph node and pancreatic metastases after curative resection of rectal cancer: a case report

**DOI:** 10.1186/s40792-016-0177-y

**Published:** 2016-05-25

**Authors:** Hitoshi Hino, Hiroyasu Kagawa, Yusuke Kinugasa, Akio Shiomi, Tomohiro Yamaguchi, Yushi Yamakawa, Masakatsu Numata, Teiichi Sugiura, Katsuhiko Uesaka

**Affiliations:** Division of Colon and Rectal Surgery, Shizuoka Cancer Center Hospital, 1007 Shimonagakubo, Nagaizumi-cho, Sunto-gun, Shizuoka 411-8777 Japan; Division of Hepato-Biliary-Pancreatic Surgery, Shizuoka Cancer Center Hospital, 1007 Shimonagakubo, Nagaizumi-cho, Sunto-gun, Shizuoka 411-8777 Japan

**Keywords:** Rectal cancer, Retroperitoneal lymph node metastasis, Pancreatic metastasis, Surgery, Recurrence

## Abstract

**Background:**

The possible benefits of the surgical resection of multiple metastases in rare sites from colorectal cancer (CRC) are still unclear. Therefore, more cases are needed to investigate the surgical outcomes of these diseases. A very rare case in which the simultaneous resection of both the metachronous retroperitoneal lymph node and pancreatic metastases from rectal cancer was successfully performed is reported.

**Case presentation:**

A 68-year-old man had undergone low anterior resection for rectal cancer. Eight months later, computed tomography showed an enlarged lymph node located below the aortic bifurcation and a pancreatic head tumor. Positron emission tomography showed increased focal uptake in these two lesions. With a diagnosis of retroperitoneal lymph node metastasis from rectal cancer and primary pancreatic cancer or pancreatic metastasis from rectal cancer, resection of the enlarged retroperitoneal lymph node and pancreaticoduodenectomy were performed. The pathological examination showed that both resected lesions were metastases from the primary rectal cancer. After the metastasectomy, the patient was given systemic chemotherapy, which was discontinued due to an adverse event. He was then followed up routinely without any medication. Sixty-nine months after the metastasectomy, he is alive without any indication of recurrence.

**Conclusions:**

Thus, even with metastases from CRC located in rare sites, an acceptable outcome can be expected following curative surgical resection in carefully selected patients. Whenever possible, an aggressive surgical approach should be included in the multimodality treatment of metastatic CRC.

## Background

Recently, the resection of metastases from colorectal cancer (CRC), such as liver and/or lung metastases, has appeared to be beneficial [1–7]. In the indications of the Japanese Society for Cancer of the Colon and Rectum Guidelines for the Treatment of Colorectal Cancer, surgical treatment is indicated for recurrent CRC limited to one organ and considered for recurrence in two or more organs, if the lesions are resectable [8]. However, most patients with multiple metastases located in sites except for the liver and lung are not candidates for resection, since they usually have widespread systemic disease at the time of diagnosis. Therefore, there are few reports about the surgical outcomes of metastatic CRC except for liver and/or lung metastases. Moreover, no prospective trials comparing the efficacy of retroperitoneal lymph node metastasectomy or pancreatic metastasectomy with non-operative management have been reported. Therefore, the possible benefits of surgical resection for these diseases have not been defined, and more cases are needed to clarify the surgical outcomes of these diseases.

This report describes a very rare case in which the simultaneous resection of both the metachronous retroperitoneal lymph node and pancreatic metastases after curative resection of rectal cancer was successfully performed. Furthermore, the patient is currently alive without any recurrence, 69 months after metastasectomy. To the best of our knowledge, this is the first case with long overall survival and disease-free survival after the resection of the retroperitoneal lymph node and pancreatic metastases from CRC.

## Case presentation

**Fig. 1:**
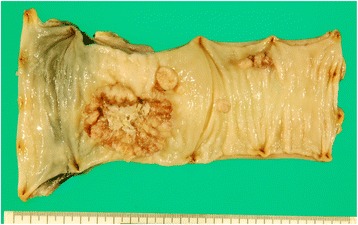
Macroscopic findings of the primary rectal cancer. A type 2 tumor (40 × 50 mm) is seen in the resected rectum

**Fig. 2:**
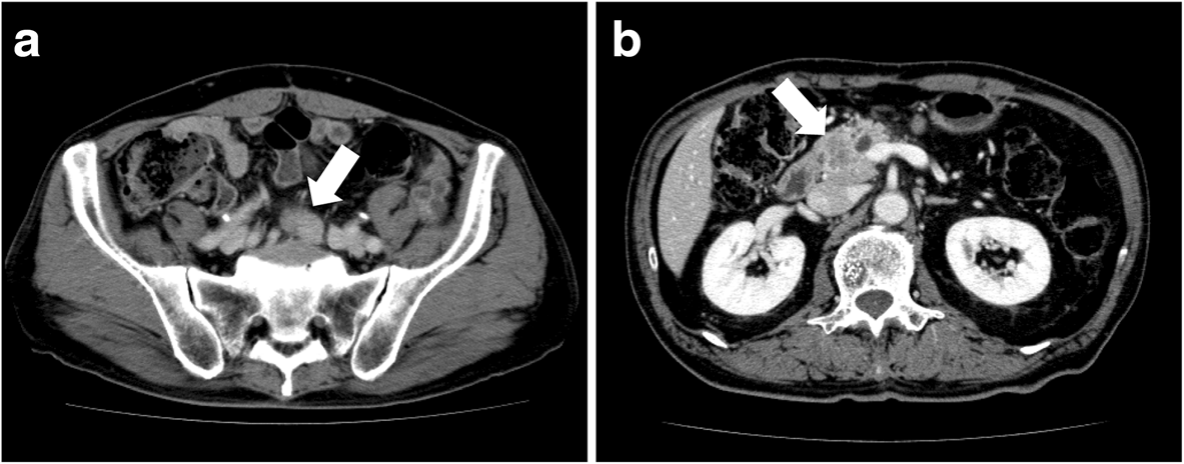
Preoperative findings of abdominal contrast CT. **a** An enlarged retroperitoneal lymph node (28 mm in diameter) is confirmed below the aortic bifurcation (*arrow*). **b** A hypovascular tumor (25 mm in diameter), which was growing invasively, is shown in the head of the pancreas (*arrow*)

**Fig. 3:**
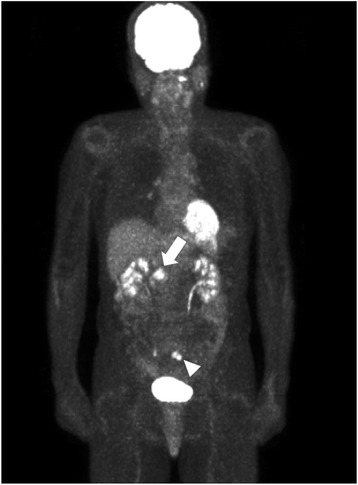
Preoperative findings of ^18^FDG-PET. The enlarged retroperitoneal lymph node has a maximum standardized uptake value (SUV max) of 6.08 (*arrow head*), and the pancreatic tumor has an SUV max of 7.83 (*arrow*)

**Fig. 4:**
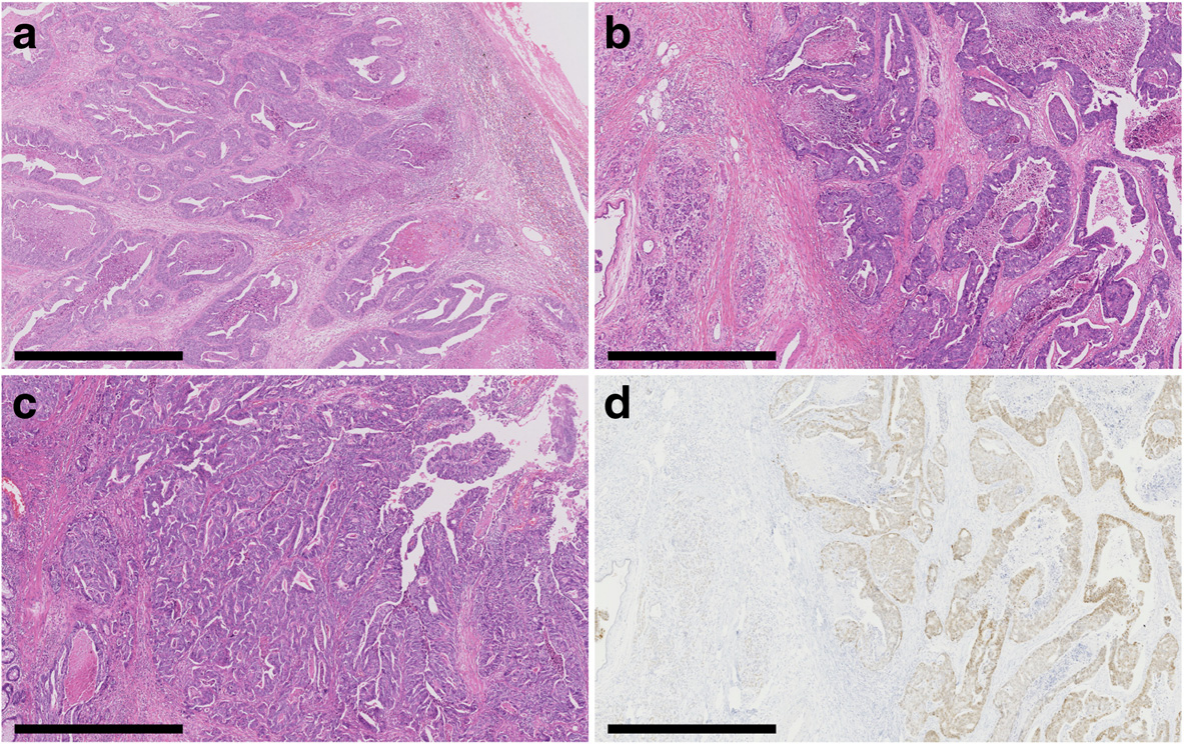
Pathological findings of resected tumors. **a** Pathological findings of the retroperitoneal lymph node (H&E). **b** Pathological findings of the pancreatic tumor (H&E). **c** Pathological findings of the primary rectal cancer (H&E). *Bar*: 1 mm. On pathology, both the retroperitoneal lymph node and pancreatic tumors show adenocarcinoma, identical to the primary rectal cancer and compatible with metastases of the rectal cancer. **d** Positive immunohistochemical staining for CDX-2 in the pancreatic tumor

A 68-year-old man had undergone low anterior resection and regional lymph node dissection for rectal cancer (Fig. 1). The pathological examination showed the tumor to be a moderately differentiated adenocarcinoma with invasion to the subserosa (T3) and regional lymph node metastasis (N1). No distant metastases were found at the time of operation (M0), and the pathological staging of the tumor was stage IIIB, according to the seventh edition of the International Union Against Cancer TNM classification. He was treated with oral uracil and tegafur plus leucovorin for 5 cycles as adjuvant chemotherapy. Eight months after the initial surgery, follow-up computed tomography (CT) showed an enlarged retroperitoneal lymph node located below the aortic bifurcation, which would be considered one of the aortic bifurcation nodes (Fig. 2a), a tumor in the head of the pancreas (Fig. 2b), and dilation of the common bile duct and main pancreatic duct. Positron emission tomography (PET) showed abnormal uptakes of ^18^F-fluorodeoxyglucose (^18^FDG) in these two lesions (Fig. 3). Biopsy of the narrowed section of the pancreatic duct showed an adenocarcinoma. However, it was difficult to identify whether the pancreatic tumor was primary or metastatic disease. Clinical examination was unremarkable. Blood tests showed continued elevation of serum bilirubin (>2.0 mg/dL). The serum carcinoembryonic antigen level was within normal limits (4.1 ng/mL), while the serum carbohydrate antigen 19-9 level was increased to 127 U/mL. Based on these findings, a diagnosis of retroperitoneal lymph node metastasis from the previously resected rectal cancer and primary pancreatic head cancer or pancreatic metastasis from rectal cancer was made. For these lesions, resection of the enlarged retroperitoneal lymph node and pancreaticoduodenectomy were performed. The pathological examination of the resected specimen showed that the histological type of both the retroperitoneal lymph node and the pancreatic head tumor was adenocarcinoma. Moreover, they were identical to the primary rectal cancer (Fig. 4a–c), and immunohistochemical study of the pancreatic tumor showed positive immunoreactivity for CDX-2 staining (Fig. 4d). Based on these findings, these lesions were diagnosed as metastases from the primary rectal cancer. These specimens had negative resection margins. The patient’s postoperative course was uneventful. After the second surgery, he was given 5-fluorouracil, leucovorin, and oxaliplatin (modified FOLFOX6) as adjuvant chemotherapy. However, the chemotherapy was discontinued after only 5 cycles due to an adverse event. He was then routinely followed up without any medication. Sixty-nine months after the metastasectomy, there has been no indication of recurrence.

## Conclusions

Currently, there is strong evidence in favor of metastasectomy for CRC in carefully selected patients [1–7]. However, most of the literature on metastasectomy for CRC pertains to the resection of liver or lung metastases and the significance of the resection of pancreatic and retroperitoneal (aortic bifurcation) lymph node metastases remains uncertain, since resectable lesions in these areas are much less common than liver and lung involvement.

Metastases to the pancreas are relatively uncommon. Among them, CRC is very rare as a primary site and accounts for 5.5–7.8 % of metastatic pancreatic malignancies [9–11]. Therefore, little is known about the therapeutic benefits of pancreatic metastasectomy in CRC [9–13]. It was previously reported that the median survival time (MST) and the 5-year overall survival (OS) were 54 months and 27 %, respectively, after pancreatic metastasectomy for CRC, although the number of patients was small [9]. These outcomes were similar to those for hepatic metastasectomy, suggesting that aggressive surgery for pancreatic metastases might be beneficial in carefully selected patients. As for the selection of patients for pancreatic metastasectomy, primary cancer type, controlling the primary site, isolated metastasis, resectability of the metastasis, and patient fitness were presented as criteria [9]. In addition, it was also suggested that, if the patient had extra-pancreatic metastasis, pancreatic metastasectomy should be an option as long as all metastatic sites could be resected. On the other hand, Sperti et al. suggested that pancreatic metastasectomy for CRC may be considered palliative treatment, and an aggressive surgical approach may be advocated in selected patients, in particular, in symptomatic patients with isolated pancreatic metastasis [10].

The reported incidence of isolated retroperitoneal lymph node metastases from CRC is 1–2 % [14–16]. As for the benefits of the surgical resection of retroperitoneal lymph node metastases from CRC, except for the reports only about paraaortic lymph node metastasis, few reports showed favorable outcomes in selected patients [17–19]. Two studies defined retroperitoneal lymph node metastases as lymph node metastases limited by the ureters laterally, iliac vessels inferiorly, and the retropancreatic area [18] or the celiac area [17] superiorly. The MST after the complete resection of retroperitoneal lymph node metastases from CRC was 53–60 months, and the 3-year OS was 63–81 %. Furthermore, these reports included patients with extra-retroperitoneal metastases, such as liver, lung, peritoneal, and inguinal lymph node metastases, and the number of metastatic sites [18] or extra-retroperitoneal metastases [17] was not significantly associated with the outcome. In addition, Shibata et al. showed that the resection of isolated retroperitoneal recurrences was significantly associated with better survival compared with exploration only (MST, 40 versus 3 months) [14]. Moreover, for patients undergoing retroperitoneal metastasectomy, a negative surgical margin and smaller tumor size (≤5 cm) predicted a better prognosis [14]. Taken together, these findings suggest that the surgical resection of retroperitoneal lymph node metastases from CRC could improve survival in selected patients, even in the presence of extra-retroperitoneal metastasis. In addition, complete resection of the metastases with negative surgical margins would be crucial to improve survival.

In the present case, the simultaneous resection of both the metachronous retroperitoneal lymph node and pancreatic metastases from rectal cancer was successfully performed. Furthermore, this patient has had prolonged survival. In a review of the English literature, no reports with detailed clinical information on patients undergoing resection of both the retroperitoneal lymph node and pancreatic metastases from CRC could be identified. To the best of our knowledge, this is the first case with long overall survival and disease-free survival after the resection of these lesions. In this case, the metastatic lesions were located in the two organs. However, both metastatic lesions could be resected completely. Moreover, good control of the primary site was achieved, patient fitness was good, and the retroperitoneal lymph node metastasis was small in size. Collectively, these factors might have contributed to the long-term survival after metastasectomy in this case.

On the other hand, the therapeutic benefits of preoperative or postoperative chemotherapy for retroperitoneal lymph node or pancreatic metastases from CRC are almost unknown. There was heterogeneity among the studies in the use of adjuvant therapy, the chemotherapeutic agents used, and so on. Therefore, it is difficult to determine whether these multimodality treatments have therapeutic benefits compared with surgery alone. However, currently, anticancer agents and molecular targeted agents have made remarkable progress, and therefore, there will be advantages of multimodality treatment for more aggressive CRC, such as disease with retroperitoneal lymph node and/or pancreatic metastases.

In conclusion, an acceptable prognosis could be expected by the potentially curative resection of metastases from CRC, even if they are located in the retroperitoneal lymph nodes and/or the pancreas, in carefully selected patients. Although the outcomes of chemotherapy for CRC have improved markedly, complete cure has not been achieved. Therefore, whenever possible, an aggressive surgical approach should be included in the multimodality treatment of metastatic CRC.

## Abbreviations

^18^FDG: ^18^F-fluorodeoxyglucose
CRC: colorectal cancer
CT: computed tomography
MST: median survival time
OS: overall survival
PET: positron emission tomography
SUV: standardized uptake value
